# Role of commensal and probiotic bacteria in human health: a focus on inflammatory bowel disease

**DOI:** 10.1186/1475-2859-12-71

**Published:** 2013-07-23

**Authors:** Rebeca Martín, Sylvie Miquel, Jonathan Ulmer, Noura Kechaou, Philippe Langella, Luis G Bermúdez-Humarán

**Affiliations:** 1 INRA, UMR1319 Micalis, Jouy-en-Josas, F-78350, France; 2 AgroParisTech, UMR Micalis, Jouy-en-Josas, F-78350, France

**Keywords:** Bacteria-host crosstalk, Dysbiosis, Genetically modified microorganisms

## Abstract

The human gut is one of the most complex ecosystems, composed of 10^13^-10^14^ microorganisms which play an important role in human health. In addition, some food products contain live bacteria which transit through our gastrointestinal tract and could exert beneficial effects on our health (known as probiotic effect). Among the numerous proposed health benefits attributed to commensal and probiotic bacteria, their capacity to interact with the host immune system is now well demonstrated. Currently, the use of recombinant lactic acid bacteria to deliver compounds of health interest is gaining importance as an extension of the probiotic concept. This review summarizes some of the recent findings and perspectives in the study of the crosstalk of both commensal and probiotic bacteria with the human host as well as the latest studies in recombinant commensal and probiotic bacteria. Our aim is to highlight the potential roles of recombinant bacteria in this ecosystem.

## Relationship humans-bacteria: a history of common benefits

The human gastrointestinal tract (GIT) is one of the most complex ecosystems known. Its microbiota consists of a large number of bacteria (10-fold more than the total number of human cells) that shapes many important physiological and metabolic processes as well as the development of the immune system [1,2]. The advances of molecular techniques have shown that the collective adult human GIT microbiota is composed of up to 1000–1150 bacterial species [3,4]. The most frequently found species are Gram-positive bacteria. The predominant species (46-58%) are those with low GC-content and the Clostridium group is the most abundant in this complex ecosystem [4]. Physiological conditions differ widely in the human GIT leading to an individual gut microbiota [5]. Some studies suggest that the faecal microbiota does not necessarily represent the bacteria inside the GIT [6-8]. Additionally, the intestinal lumen microbiota differs significantly from the one in the mucus layer and near the epithelium due to the poor accessibility of the crypts covered by mucins [9].

Despite its complexity, the faecal microbiota of adult human individuals is unique and highly stable through time [10]. Primocolonizing microorganisms appear in the gut immediately after birth in an organized and lifelong process [11]. During the first year, after the initial establishment of the intestinal microbiota, its composition is relatively simple and varies between individuals [12]. After that, the main dominant groups of the adult microbiota will be conserved between all individuals. These groups are stable in spite of the great number of factors that can affect them [13]. In contrast, at the phylum level, the variation is higher between individuals although people who are related tend to have similar microbiota perhaps due to a shared environment and genetic similarities [13].

**Figure 1:**
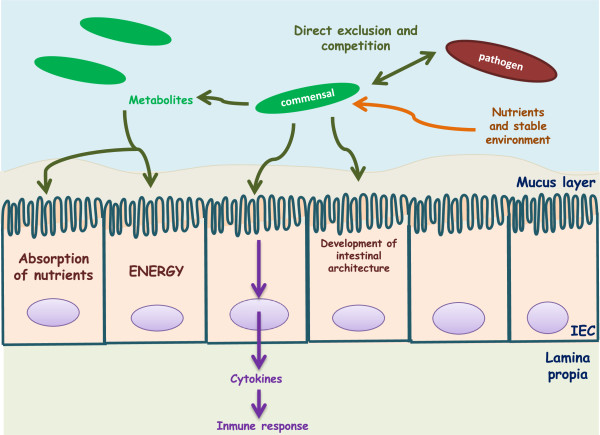
**Commensal bacteria cross talk with the host.** Commensal bacteria supply the host with essential nutrients and defend the host against opportunistic pathogens. They are involved in the development of the intestinal architecture and immunomodulatory processes. On the other hand, the host provides the bacteria with nutrients and a stable environment.

This microecosystem, which is a direct consequence of the mutualism between the host and its microbiota, is fundamental for the maintenance of the homeostasis of a healthy individual [5]. Commensal bacteria provide the host with essential nutrients. They metabolize indigestible compounds, defend against colonization of opportunistic pathogens and contribute to the development of the intestinal architecture as well as stimulation of the immune system among others [11]. Conversely, the host provides the bacteria with nutrients and a stable environment [5] (Figure 1). Both host and indigenous microorganisms have then adapted to each other in a particular case of microevolution to maintain the benefits that this mutualism confers [2].

## Probiotics: an additional bacterial advantage for humans

Probiotics are defined as “live microorganisms which when administered in adequate amounts confer a health benefit on the host” [14]. This concept is based on the observations made by Élie Metchnikoff in 1907 in which the regular consumption of Lactic Acid Bacteria (LAB) in fermented dairy products, such as yogurt, was associated with enhanced health and longevity in many people living in Bulgarian villages [15].

Most probiotics belong to LAB, but new species and genera are being assessed for future use. Some other well-known probiotics are *Bifidobacterium* sp., one strain of the Gram-negative bacterium *Escherichia coli* Nissle 1917 and the yeast *Saccharomyces boulardii*[16].

However, not all microorganisms are beneficial and screenings in order to identify novel candidate probiotics with immunomodulatory properties are frequently performed [17]. In fact, a Dutch trial using a probiotic preparation in patients with acute pancreatitis showed to be harmful [18]. These negative results show how important it is to choose the right protocol (indeed, the main problem with this study was the bad idea to administer massive quantitites of probiotics in very vulnerable patients) and to make a careful selection when using probiotics in humans. Certainly, a supposed beneficial effect of a specific strain cannot be extrapolated to another strain even within to the same species [19,20].

Modulation of host immunity and promotion of host defense are the most commonly supported benefits of the consumption of probiotics. For a microorganism to be considered probiotic (*i.e.* bacteria or yeast), the following criteria need to be fulfilled: i) it should have a clear beneficial effect on the host, ii) it should be non-pathogenic, iii) it should be able to survive transit through the GIT and iv) a large number of viable bacteria must be able to survive prolonged periods (*i.e.* upon storage) [21].

## What happens if homeostasis is broken?

The term dysbiosis refers to microbial imbalances on or within the body. When homeostasis (balanced microbial ecosystem) is broken, different subdominant opportunistic bacteria can grow leading to a situation of illness. Additionally, when commensal bacteria are depleted, an abnormal health situation can be triggered due to a lack of the benefits these bacteria provide rather than the overgrowth of pathobionts.

a) Binomial dysbiosis – illness

The term dysbiosis has been related to many different kinds of pathologies although it is not clear whether the imbalance of microbiota is a cause or a consequence of the illness. The clearest correlation between dysbiosis and disease has been found with inflammatory bowel diseases (IBD) where the proportion of Firmicutes, in particular *Faecalibacterium prausnitzii*, was found to be low in patients that exhibited endoscopic recurrence 6 months after surgery [22,23]. IBD, including Crohn’s disease (CD) and ulcerative colitis (UC), are characterized by an abnormal activation of the immune system associated with the gut, resulting in a chronic inflammation of the digestive system. However, some other factors also seem to be involved in IBD such as genetic components [24,25], immunological disorders [26], environmental factors [27,28], pathogens [29,30], and microbiota [10]. Faecal analyses have shown a quantitative and qualitative reduction in the representation of the Firmicutes phylum, mostly the clostridial cluster IV members in CD patients while low numbers of total lactobacilli have been reported in UC members [31,32], although no correlation was found between *F. prausnitzii* abundance and the severity of CD [33].

Even if the composition of the human microbiota is different in each individual, changes in phylogenic distribution have also been specifically found in obese and diabetic individuals versus normal ones [34,35] (Table 1). The importance of the human microbiota has been demonstrated in the hygiene hypothesis, defined in 1989 by Strachan [36] who postulated that low exposure to infectious agents in early life explains the increased numbers of people suffering from allergies and asthma in developed countries. This hypothesis suggests that a well-balanced human microbiota is a factor that protects from such pathologies [37,38].

Some microbial activities have shown relevance to health and disease. Following this line of thought, the production of short chain fatty acids (SCFA) such as butyrate has been proposed to protect against different illnesses (Table 2).

b) Probiotics to restore dysbiosis

As we have seen before, dysbiosis are involved in a great variety of different illnesses. Considering this fact, the administration of beneficial microorganisms to restore the normal ecosystem is a strategy to improve the health status of the patient and/or to prevent a normal healthy individual from acquiring dysbiosis in the future. Currently, there is evidence of the use of probiotics as therapeutics against traveler’s diarrhea, irritable bowel syndrome (IBS), IBD, lactose intolerance, peptic ulcers, allergy and autoimmune disorders among others [55-60]. For instance, it has been suggested that colonization of the GIT with Bifidobacteria properly shapes gut microbiota, induces oral tolerance and decreases the frequency of allergic disorders [61]. For lactobacilli, a clinical study demonstrates that perinatal administration of a probiotic strain of *Lactobacillus rhamnosus* GG (LGG) reduces the development of atopic eczema in children [61-64]. This effect may be due to the anti-inflammatory properties of this probiotic bacterium. Consumption of LGG by children with atopic dermatitis has been reported to enhance the production of the anti-inflammatory cytokine IL-10 [65]. Other studies have demonstrated that oral administration of *L. casei* Shirota strain to mice inhibited specific IgE production [66] while Abrahamsson et al. [67] have observed in a double-blind, randomized, placebo-controlled trial that infants treated with *L. reuteri* ATCC 55730 strain had less IgE-associated eczema.

As mentioned above, when the equilibrium of the microbiota is disturbed, the bacterial ecosystem is thought to contribute to several intestinal diseases such as IBD. In fact, gut microbiota metabolize nutrients, produce vitamins and degrade toxic products (such as: carcinogens, food additives, bile salts, and cholesterol, among others) [68,69], where the importance and interest in modulate microbiota with probiotic products. Results from animal models and human clinical trials have confirmed various therapeutic effects of selected strains of probiotics in IBD [56,70]. For instance, *L. casei* BL23 strain has shown anti-inflammatory effects in a murine DSS-induced colitis model [71] and some other probiotic strains (*eg.* VSL#3, LGG, BIFICO, *E. coli* Nissle) have shown effects in human patients with pouchitis, UC and CD [72]. Additionally, the modulatory effect of *Lactobacillus acidophilus* in intestinal pain due to the induction of opioid and cannabinoid receptors has been reported in rats [73]. This fact highlights the use of probiotics in other intestinal disorders such as such as IBS, characterized by chronic abdominal pain, discomfort, bloating and alteration of bowel habits. In this sense, in the last years, many studies confirm the overall positive results of probiotics in human IBS patients using different bacteria such as: *Lactobacillus* spp.*, Bifidobacterium* and *Streptococcus* among others [74].

From this perspective, the use of probiotics has been expanded in the last few years, with a great number of probiotic products in our supermarkets and pharmacies. However, the knowledge of their mechanisms of action remains largely unknown.

**Table 1:** Some examples of disbiosis found in obesity and diabetes

**Disease**	**Disbiosis**	**Model**	**Technique**	**Sample**	**N**	**Reference**
Obesity	↓ *Bacteroidetes*	Mice C57BL/6J	16S RNA sequencing	Distal intestinal content	5088 sequences	[39]
	*↑ Firmicutes*					
	↑ *Firmicutes*	Humans	16S RNA sequencing	Faecal	12	[40]
			Real time PCR		40	[41]
	*↑ Bacteroidetes*					
			16S RNA sequencing		154	[42]
	↑ H2-producing bacterial groups (*Prevotellaceae* family and certain groups of *Firmicutes*)	Humans	16S RNA sequencing	Faecal	9	[43]
Type 1 diabetes	Ratio bacteriodietes/firmicutes altered	Non obese diabetic mice (NOD)	16S RNA sequencing	Faecal		[44]
Type 2 diabetes	↓ *Prevotella,*	Humans	16S RNA sequencing Real time PCR	Faecal	36	[45]
	*↓ Bifidobacterium spp*					
	*↓ F. prausnitzii*					
			16S RNA sequencing Real time PCR DGGE		28	[46]
	*↑ Bacteroides*					

**Table 2:** Benefical effects of short chain fatty accids (SCFA)

**SCFA**	**Model**	**Effect**	**Reference**
Butyrate	Tumorigenesis in rat colon and Human colonic cells	Inhibit the genotoxic activity of nitrosamides and hydrogen peroxide	[47]
	Human adenocarcinoma R6/C2 and AA/C1 cells and carcionoma PC/JW/F1 cells	Induce apoptosis	[48]
	Human intestinal primary epithelial cells (HIPEC), HT-29 and Caco-2 cells	Immunoregulatory effects	[49]
	Humans with distal ulcerative colitis	Improves UC symthoms	[50]
Butyrate/acetate/propionate	Humans with diversion colitis	Improves the macroscopic and histological signs of inflammation	[51]
Propionate	HT-29 cells	Anti-proliferative effects	[52]
	Madin-Darby bovine kidney epithelial cells (MDBK)		[53]
Acetate	*E. coli* O157:H7 infection	Protection	[54]

## New prospects of commensal and probiotic bacteria: genetically modified microorganisms

a) Strategies to deliver therapeutic molecules.

**Figure 2:**
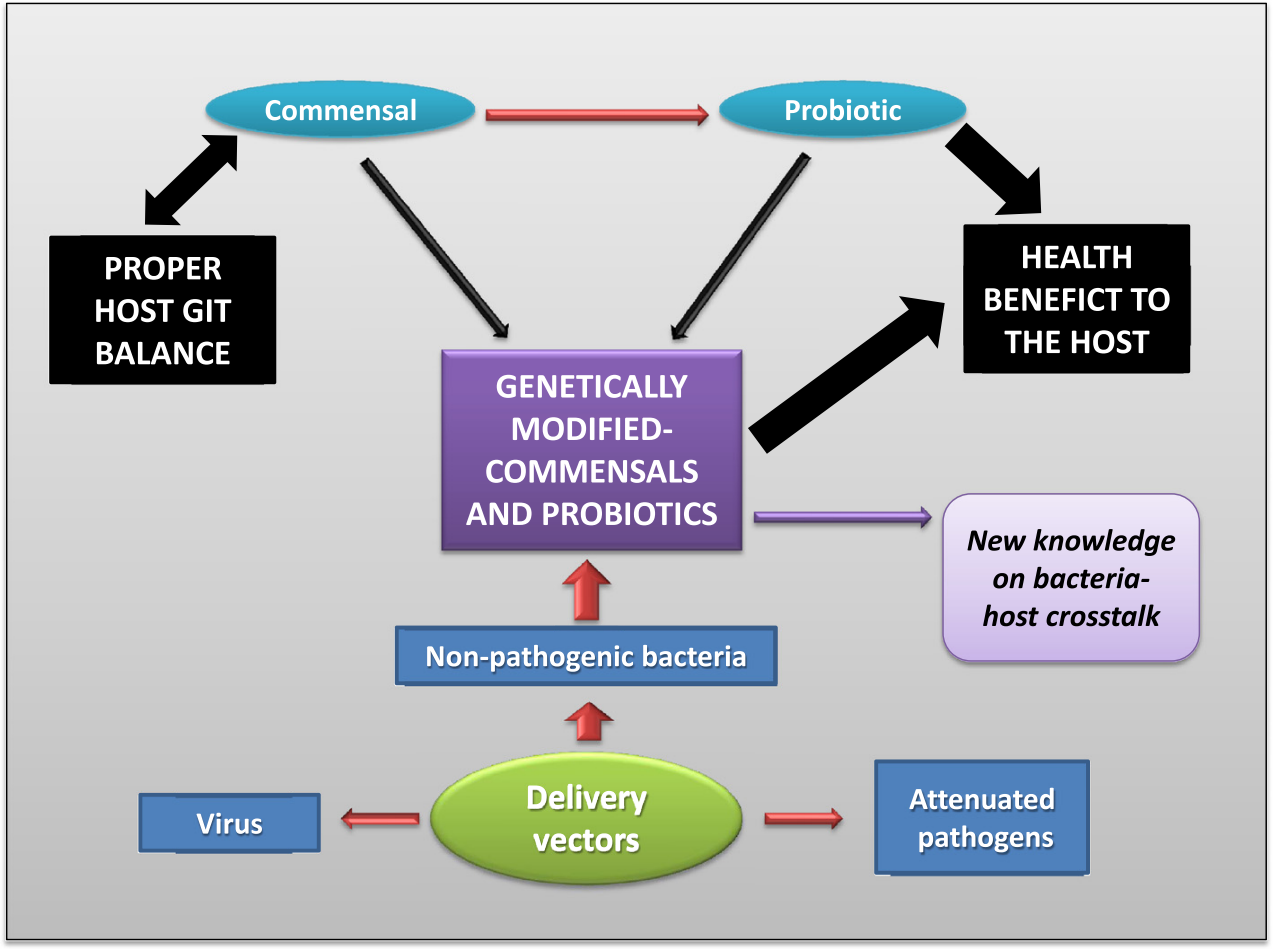
**Extension of the commensals-probiotics hypothesis.** The development of a genetically modified commensal (or probiotic) could reach all the beneficial properties found in a commensal bacterium joint to the probiotic effects due to the ability to deliver molecules to the gastrointestinal tract (GIT).

Since probiotic therapy is mainly focused on restoring the normal balance of the intestinal ecosystem, we can deduce that the use of commensal bacteria as probiotics is the natural way to get rid of dysbiosis within the GIT. Furthermore, they represent potential live delivery vectors for target compounds (Figure 2). The use of live delivery vectors, such as food-grade LAB, at the mucosal level has been widely described before [75-79]. They are based on the use of recombinant bacteria producing the heterologous molecule of interest *in vivo* and their use is mainly related to vaccines. This is due to the fact that genetically engineered bacteria or viruses (used as carriers) producing heterologous antigens can increase the immunogenicity of otherwise weakly immunogenic antigens. In addition, such live bacterial vectors have the additional advantage (compared to viruses) of having a genome able to harbor many heterologous genes, in contrast to viruses where the capacity to encapsulate foreign DNA is limited. This research field comes from exploring novel effective strategies to deliver therapeutic molecules to the mucosal tissues in order to avoid degradation and promote uptake of the antigen *in situ* (*ie.* in the GIT), and stimulate adaptive immune responses rather than the tolerogenic immune responses that are observed in feeding studies with soluble antigens [80]. Besides the enhancement of the potency and specificity of mucosal delivery of therapeutic molecules, the use of mucosal routes reduces potential side effects observed in systemic ones.

In principle, two types of bacterial vectors can be used to deliver compounds at the mucosal level: attenuated pathogens and non-pathogenic bacteria. Gram-positive commensal or food-grade bacteria constitute attractive good alternatives to pathogenic bacteria [79,81]. Particularly, the food-grade LAB are attractive candidates because they have been used for centuries in the fermentation and preservation of food, and are considered to be safe organisms with a GRAS (Generally Recognized As Safe) status. One major advantage of LAB as delivery vectors for vaccines purposes is their potential to elicit both antigen-specific immune responses at mucosal surfaces and effective systemic immune responses. Indeed, some studies have successfully shown that candidate LAB vaccines elicited antigen-specific IgA responses in feces, saliva or bronchoalveolar and intestinal lavage fluids, as well as antigen-specific IgA-secreting cells in the lungs and mesenteric lymph nodes [82-94].

These studies with encouraging results confirm the potential use of LAB as live vectors for mucosal immunization and/or therapy. In this context, the model LAB species, *Lactococcus lactis,* has been used for the heterologous expression of therapeutic proteins such as: antigens, cytokines and enzymes. Thus, the resulting recombinant lactococci strains have been successfully tested for their prophylactic and therapeutic effects in many animal models such as: Human Papillomavirus type-16 (HPV-16)-induced tumors in mice [83,88], bovine β-lactoglobulin (BLG)-allergic reaction in mice [95,96] and body weight and food consumption in obese mice [85,97].

b) Use of recombinant LAB and commensals in IBD.

Modulation of the mucosal immune system has been demonstrated in IBD. The currently used therapies to treat IBD are based on anti-inflammatory drugs combined with immunosuppressives [98]. Immunomodulatory molecules have been efficient in the control of inflammation and in the remission of the episodes of the illness. The use of LAB to prevent and treat colitis was performed with a recombinant *L. lactis* strain producing and delivering IL-10, an anti-inflammatory cytokine, *in situ* in different mouse models [99]. Daily mucosal administration of recombinant *L. lactis* secreting IL-10 led to a 50% decrease of the colitis induced by the administration of dextran sodium sulfate (DSS) in mice. This beneficial effect was dependent on the *in situ* secretion of IL-10 by the live lactococci. The rationale in using recombinant *L. lactis* for a local delivery of IL-10 in IBD is due to the large number of scientific studies proving that the topical treatment with this cytokine has clinic benefits [100] although systemic IL-10 administration in Crohn’s disease patients has been associated with considerable side effects which are partly due to the fact that systemic IL-10 induces the pro-inflammatory cytokine IFN-γ [101-103]. To address the safety issues in using genetically modified organisms in humans, the gene encoding the *L. lactis* thymidylate synthase (*thyA*) was replaced by the gene of the human IL-10 (hIL-10) to generate one *L. lactis* strain auxotrophic for thymidine or thymine [104]. The viability and contention of this strain was validated *in vivo* in a swine model [104]. Additionally, a small Phase I clinical study in patients with CD using the thyA-/hIL-10+ strain was recently conducted. This study not only showed that the contention strategy is effective, but also that the mucosal expression of IL-10 by *L. lactis* is feasible in humans [105]. However, a phase IIA trial was performed which revealed that, although safety, tolerability, environmental containment and assessment of biomarkers associated with the strains have been achieved, no statistically significant difference has been found versus placebo in terms of beneficial effects (press release published in 2009). Due to these results, the necessity to optimize the LAB delivery strategy (new strains, different expression systems and different nature of the delivered molecules) is a requirement to reach a clear demonstration of their efficacy in human clinical trials, leading to their better acceptance [76-78,106-109]. The delivery of immunosuppressive cytokines by other bacteria has also been tested. The probiotic potential of IL-10-expressing *E. coli* Nissle 1917 has been outlined in a mouse model of IBD [110]. The use of LAB to produce Trefoil Factors (TFF) at the mucosal level to treat IBD has also been studied. TFF are a class of nonmitogenic peptides that play important roles in the protection and repair of the intestinal epithelium [111]. These peptides are known because of their strong protective effects and for repairing the mucosa after damage. For this reason, they are interesting molecules to potentially treat IBD. However, when they are administered by an oral route, they adhere to the gut mucosa and are absorbed at the intestinal level. Interestingly, intragastric administration of recombinant *L. lactis* secreting TFF leads to the expression of the active peptides in the colon and prevents and repairs the damage due to the acute colitis induced by DSS [112]. Another strategy to treat colitis was also based on the use of recombinant *L. lactis* secreting the LcrV antigen, an anti-inflammatory protein produced by *Yersinia pseudotuberculosis* to escape the immune response of the host. The therapeutic and protective potential of this strain was evaluated using two colitis murine models: DSS and trinitrobenzene sulfonic acid (TNBS) [113]. Still, another strategy to treat IBD is the use of antioxidant enzymes. It is well established that GIT inflammation is associated with an influx of neutrophils and macrophages and with the production of inflammatory mediators such as: proteases, cytokines and reactive oxygen species (ROS) [114]. ROS include the superoxide radical (O_2_°-), hydrogen peroxide (H_2_O_2_), and the hydroxyl radical (·OH). Their reactivity toward lipids, proteins and DNA causes both cytotoxic and mutagenic cellular damages [115]. To detoxify ROS, cells have evolved protective mechanisms *via* antioxidant enzymes such as superoxide dismutases (SOD) and catalases (CAT) which degrade O_2_°^-^ and H_2_O_2_ respectively, and thus prevent the formation of·OH [116]. In this context, different studies have shown that recombinant strains of *Lactobacillus* spp. expressing either SOD or CAT can reduce inflammation in mouse models [71,117-119]. In some cases, the anti-inflammatory mechanism of these recombinant strains has been elucidated and linked to the inhibition of neutrophil recruitment [117]. The mucosal expression of elafin, a natural protease inhibitor expressed in healthy intestinal mucosa, is diminished in patients with IBD. Recently, LAB secreting elafin have been tested in chronic and acute colitis models and the inflamed epithelium was protected from increased intestinal permeability and from the release of cytokines and chemokines by LAB secreting elafin [120].

In addition to these recombinant probiotic strains, mutants in specific genes encoding potential probiotic functions (mucal adhesion factors, resistance to acid, specific cell wall components, etc.) have been engineered to compare their biological effects with that of their wild-type counterparts. In 2005, Grangette et al. [121] has provided an illustration of this approach by constructing a mutant of *Lactobacillus plantarum* impaired in its capacity to incorporate D-alanine in teichoic acids (Dlt^-^ mutant) [121]. Strikingly, when tested *in vivo*, the Dlt^-^ mutant proved to be more protective in a mouse model of colitis than the wild-type strain. Furthermore, the use of lipoteichoic acid (LTA)-deficient *L. acidophilus* in *in vitro* analysis enhances IL-10 production by dendritic cells and macrophages and downregulates IL-12 and TNF-α [122]. This strain is also able to significantly protect dextran sulfate sodium and CD4^+^CD45RB^high^ T cell-induced colitis in mice [123] and to normalize innate and adaptative pathogenic immune responses in established colonic polyps causing cancer regression [124]. However, no protective effects have been found in LTA-induced pro-inflammatory signals and subsequent colitis [125]. Altogether, with the possibility to express different factors such as ScFv antibodies, host targeting molecules and immunomodulators in LAB, more applications and progress towards studies in humans should be performed in a next future.

The next step in this field seems to choose new delivery bacteria. As mentioned above, *L. lactis* is the most widely used LAB in the production of heterologous proteins and is considered as the model LAB and live delivery vector model [75]. Despite all the advantages mentioned above, this bacterium has a short survival time (~24 hours) in the human GIT leading thus to a reduced time of action. In contrast, other probiotic bacteria which could be natural colonizers of the GIT, could combine their intrinsic probiotic effects to the probiotic effects conferred by the heterologous delivered protein. The most achieved project in this field is the one based on the use of the commensal *Bacteroides ovatus*[126] Indeed, Hamady et al. [127] chose this bacterium for the *in vivo* delivery of proteins for its ability to colonize the colon and its xylan utilisation properties. They were able to develop a xylan-regulated delivery of i) human keratynocyte growth factor-2 to the inflamed colon [128] and ii) the human TGF-b1 to treat colitis in mice [129]. These promising results confirm the potential of the use of recombinant commensal for *in vivo* delivery.

## Conclusions and future research

We can conclude that current probiotic research encourages the search and characterization of gut bacteria as a model for finding new natural or engineered probiotic strains to be used to restore the normal balance of the human gut ecosystem.

The fact that commensal and probiotic bacteria interact with the host immune system is now well accepted and illustrated by *in vitro* and *in vivo* experiments. However, the current knowledge of the molecular mechanisms involved in this cross-talk remain poorly understood. Although some mechanisms and active compounds have been identified in a few commensal or probiotic strains [130], and taking into account that the human GIT is composed of 10^13^-10^14^ microorganisms, it is necessary to explore profoundly this research area and in particular to elucidate the exact role of bacterial compounds in homeostasis and immune response.

As for the use of genetically modified commensal and probiotic bacteria in humans, it is certain that most of the studies being done are Proof-of-Concept. However, although some researchers have claimed that genetically modified probiotics should be banned [131], the data obtained in the phase I clinical trial with the recombinant strain of *L. lactis* secreting IL-10 (see above in the text) showed that the containment strategy (*ie.* release of such genetically modified organisms into nature) used to construct the strain [104] was not only safe and effective but also that mucosal delivery of IL-10 by a genetically modified LAB is feasible in humans [105].

In conclusion, it is clear that the analysis of the impact of commensals and probiotics on the host immune system has entered a new and fascinating phase of research. This new area offers us new knowledge that can be exploited to develop new approaches to modulate host immunity for protection against infectious diseases or for immunotherapy.

## Abbreviations

BLG: β-lactoglobulin; CAT: Catalases; CD: Crohn’s disease; DSS: Dextran sodium sulfate; GIT: Gastrointestinal tract; GMO: Genetically modified organisms; GRAS: Generally recognized as safe; HPV-16: Human Papillomavirus type-16; IBD: Inflammatory bowel disease; IBS: Irritable bowel syndrome; LAB: Lactic acid bacteria; LGG: *Lactobacillus rhamnosus* GG; ROS: Reactive oxygen species; SCFA: Short chain fatty acids; SOD: Superoxide dismutases; TFF: Treefoil factors; TNBS: Trinitrobenzene sulfonic acid; UC: Ulcerative colitis

## Competing interests

The authors declare that they have no competing interests.

## Authors’ contribution

RM and LBH wrote the main text, figures and tables. SM, NK, JU, PL corrected and helped to draft the manuscript. All authors have read and approved the final manuscript.
